# Supplementary material for: The Human Leukocyte Antigen Locus and Rheumatic Heart Disease Susceptibility in South Asians and Europeans
Source: Sci Rep. 2020 Jun 2;10:9004. doi: 10.1038/s41598-020-65855-8 (PMC7265443; doi:10.1038/s41598-020-65855-8)
Supplement: Supplementary file 1 — Supplementary Information. [file 41598_2020_65855_MOESM1_ESM.pdf]

Supplementary Information

The Human Leukocyte Antigen Locus and Rheumatic Heart Disease Susceptibility in South Asians and Europeans

Kathryn Auckland[1], Balraj Mittal[2], Benjamin J. Cairns[3], Naveen Garg[4], Surendra Kumar[5], Alexander J. Mentzer[1], Joseph Kado[6], Mai Ling Perman[6], Andrew C. Steer[7], Adrian V. S. Hill[1], Tom Parks[1,8,*]

[1] The Wellcome Centre for Human Genetics, University of Oxford, Oxford, Oxfordshire, OX3 7BN, UK

[2] Department of Biotechnology, Babasaheb Bhimrao Ambedkar University, Lucknow 226025, Uttar Pradesh, India

[3] MRC Population Health Research Unit, Clinical Trial Service Unit and Epidemiological Studies Unit, Nuffield Department of Population Health, University of Oxford, Oxford, Oxfordshire, OX3 7LF, UK

[4] Department of Cardiology, Sanjay Gandhi Postgraduate Institute of Medical Sciences, Lucknow 226014, Uttar Pradesh, India

[5] Department of Cytogenetics/Anatomy, All India Institute of Medical Sciences, New Delhi 110029, Delhi, India

[6] Department of Medical Science, Fiji National University, Suva, PO Box 7222, Viti Levu, Fiji

[7] Tropical Infectious Diseases, Murdoch Children's Research Institute, Melbourne, Victoria 3052, Australia

[8] Department of Clinical Research, London School of Hygiene & Tropical Medicine, London, Greater London, WC1E 7HT, UK

Corresponding author:

*tom.parks@well.ox.ac.uk

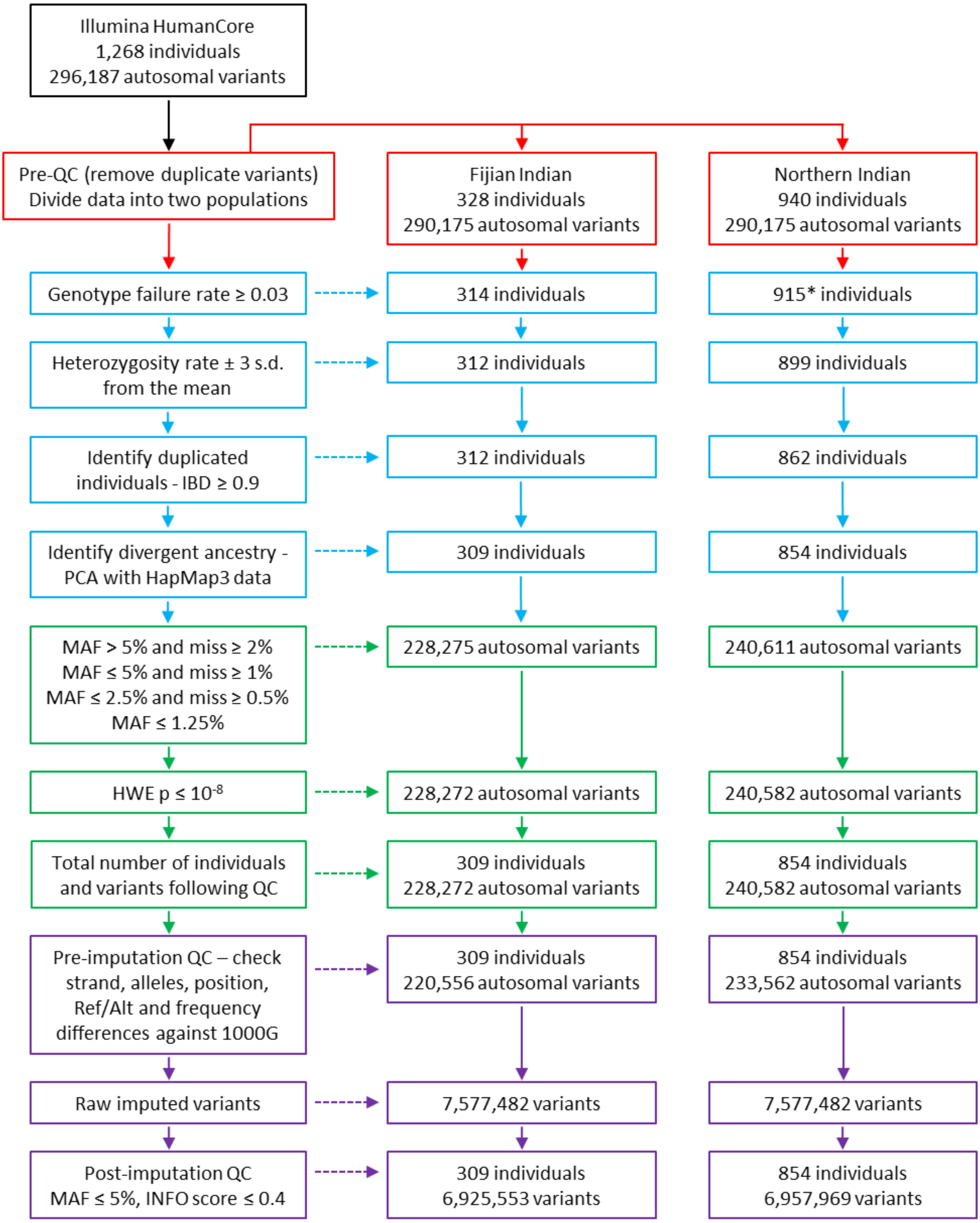

Supplementary Figure S1 | Flowchart of the GWAS quality control (QC) process both pre- and post-imputation. Red boxes denote pre-QC filtering; for the purposes of QC and downstream analyses, the data was divided by ethnicity into two populations (Fijian Indian and Northern Indian). Blue boxes denote individual QC, whereas green boxes denote variant QC. Purple boxes denote pre-imputation and post-imputation filtering. IBD, identity by descent; PCA, principal component analysis; MAF, minor allele frequency; HWE, Hardy-Weinberg Equilibrium; Ref/Alt, reference/alternative allele assignments; INFO, imputation information metric.

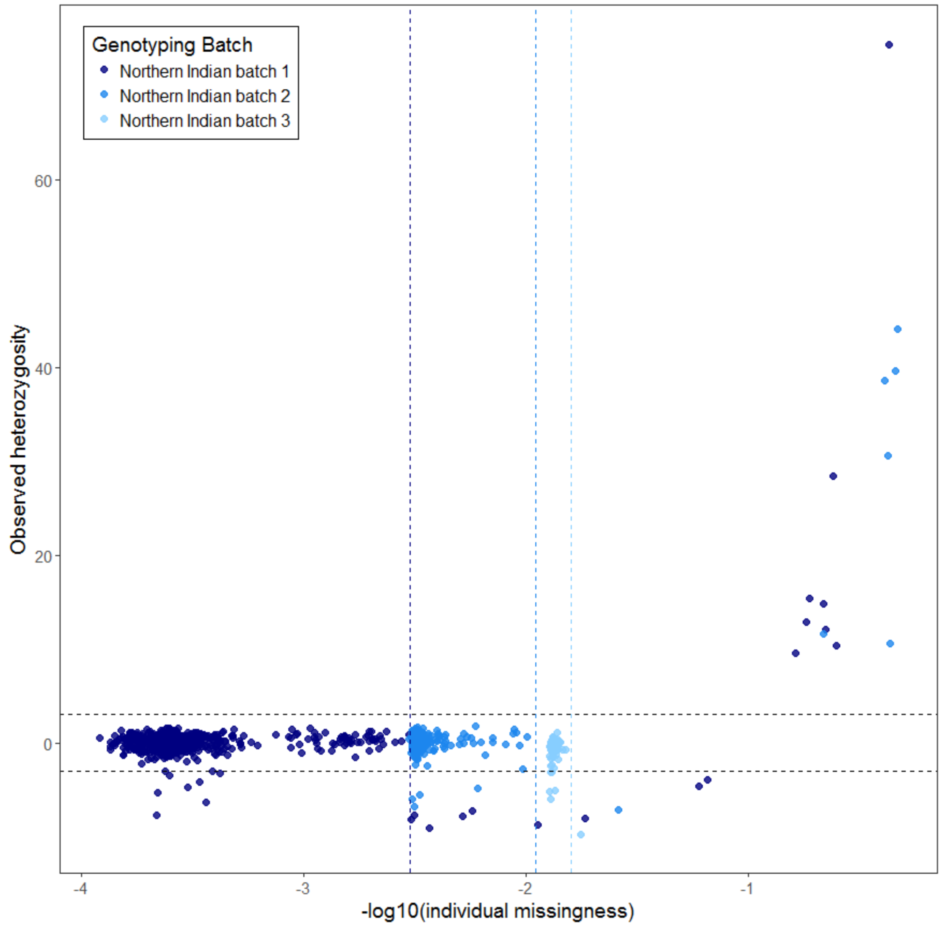

Supplementary Figure S2 | Genotype failure rate versus heterozygosity within the Northern Indian individuals. Observed autosomal heterozygosity rate per individual is plotted against the proportion of missing SNPs per individual on a logarithmic scale, coloured by genotyping batch. Horizontal lines are drawn at three standard deviations above and below the mean of autosomal heterozygosity; vertical lines are drawn at genotype failure rate ≥ 0.003 (batch 1), ≥ 0.011 (batch 2) and ≥ 0.016 (batch 3).

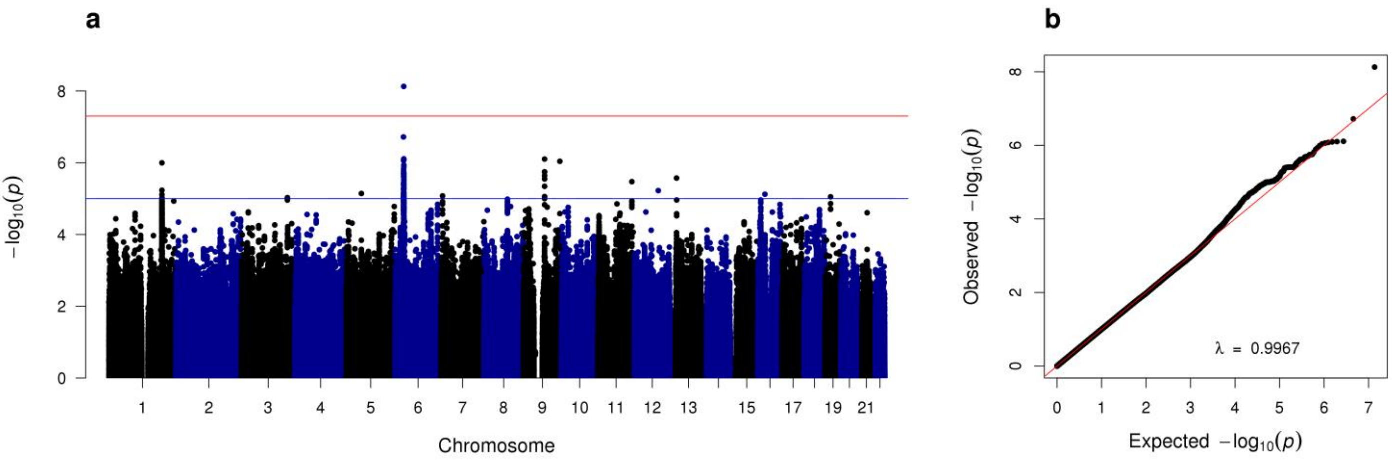

Supplementary Figure S3 | Genome-wide meta-analysis for RHD susceptibility within two South Asian populations. (**a**) For each variant, the negative common logarithm of the *P* value from an inverse-variance-weighted fixed effects meta-analysis is plotted against genomic position. The blue horizontal line indicates suggestive significance (FE meta-analysis, $P = 5 \times 10^{-5}$) and the red horizontal line indicates genome-wide significance (FE meta-analysis, $P = 5 \times 10^{-8}$). (**b**) Quantile-quantile (QQ) plot for the meta-analysis; each point represents an individual variant. An estimate of the genomic inflation factor (λ) is shown.

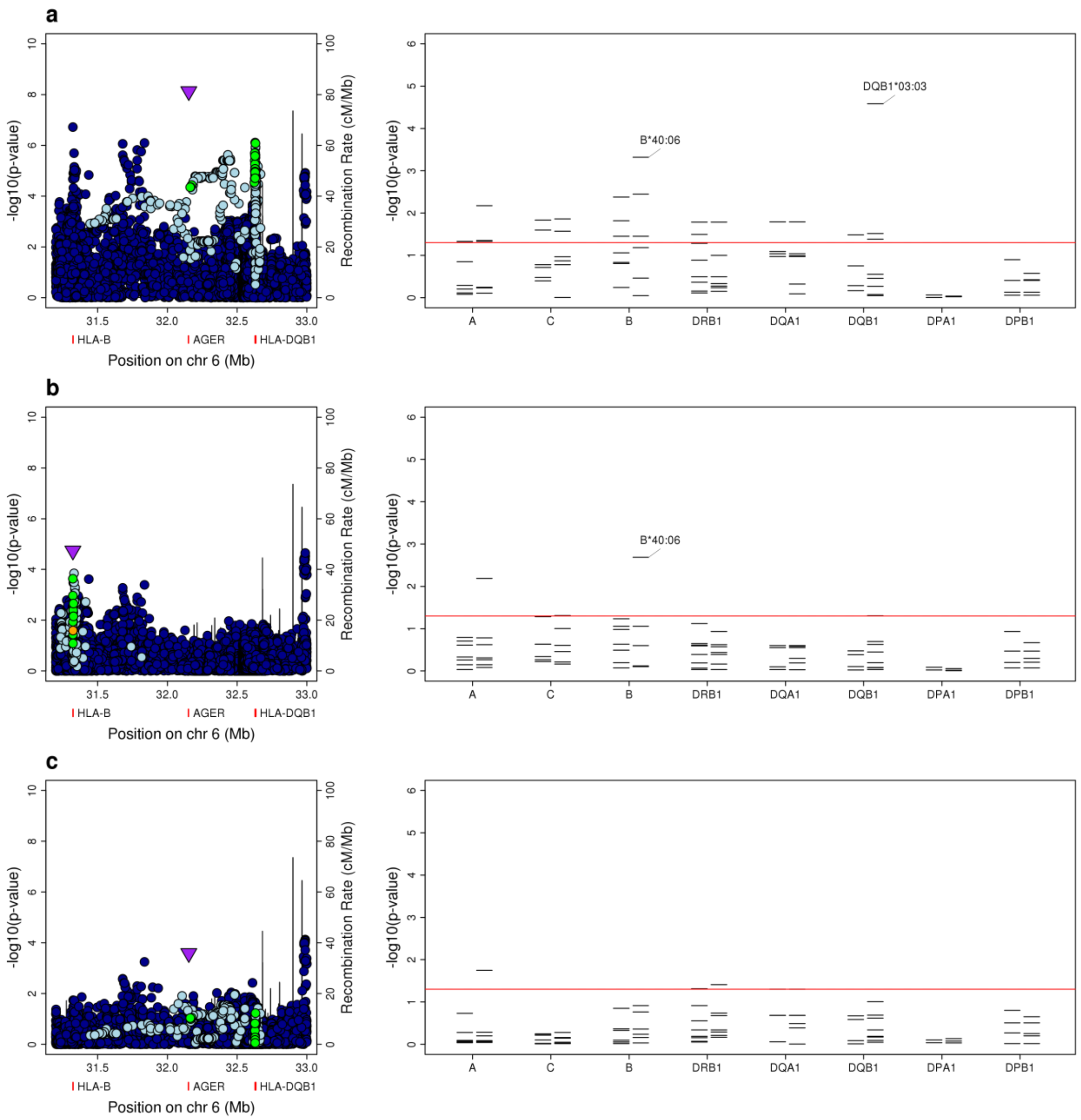

Supplementary Figure S4 | Meta-analysis of the Fijian Indian and Northern Indian data following conditional analysis. (**a**) Unconditioned analysis. (**b**) Conditioned on the top SNP (rs201026476). (**c**) Conditioned on the top class I and class II SNPs (rs3819306 and rs28724238, respectively). Left images: For the HLA region, genomic position is plotted against the negative common logarithm of the *P* value from meta-analysis. The top class I (**b**) or class III SNP (**a, c**) following meta-analysis is shown by a purple triangle. Variants are coloured by linkage disequilibrium (LD), with the most associated variant averaged across the entire dataset (estimated r2: dark blue, 0-0.2; light blue, 0.2-0.4; green, 0.4-0.6; orange, 0.6-0.8; red, 0.8-1.0). The location of *HLA-B*, *HLA-DQB1* and *AGER* are indicated by red rectangles below the x axis. The recombination rate is shown as a line plotted on the right-hand y-axis. These plots are based on those drawn by the widely used LocusZoom software. Right images: For each locus, the negative common logarithm of the *P* value from LMM analysis is plotted with two-digit alleles to the left and four-digit alleles to the right defined by HLA imputation using SNP2HLA software with the T1DGC reference panel.

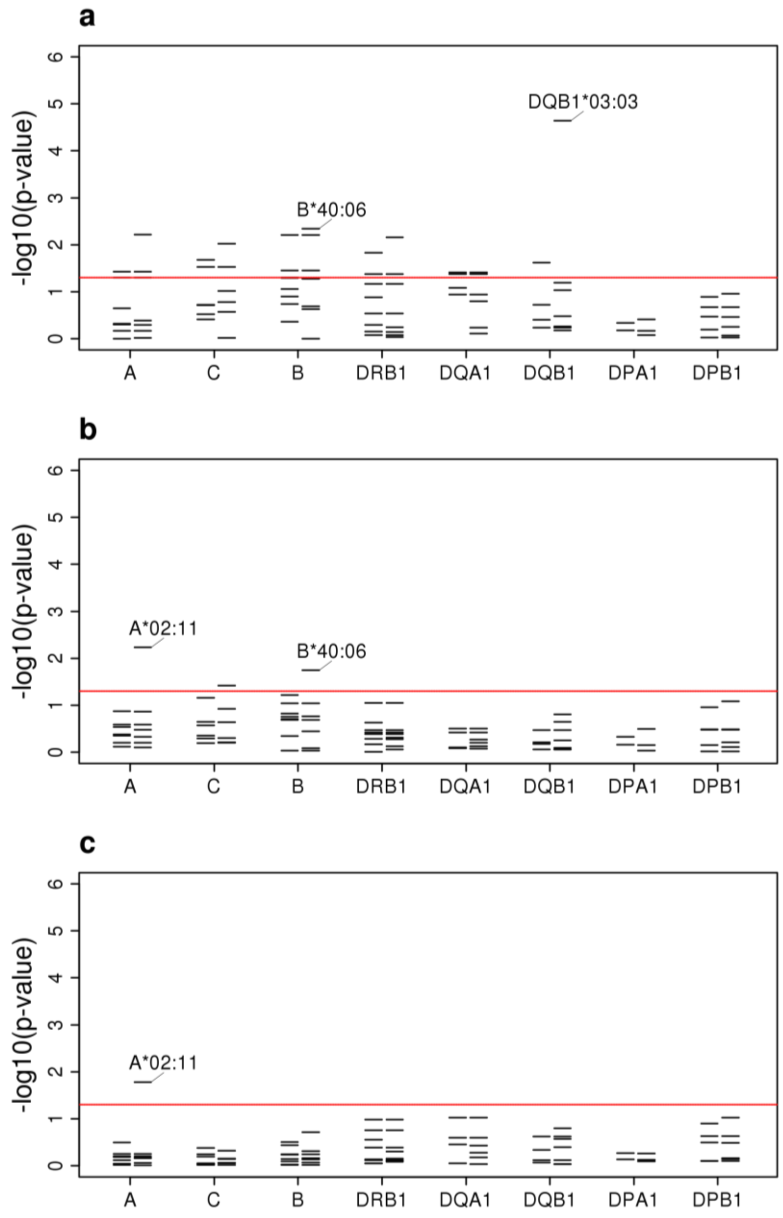

Supplementary Figure S5 | Meta-analysis of the Fijian Indian and Northern Indian data following conditional analysis. (**a**) Unconditioned analysis. (**b**) Conditioned on the top SNP (rs201026476). (**c**) Conditioned on the top class I and class II SNPs (rs3819306 and rs28724238, respectively). For each locus, the negative common logarithm of the *P* value from LMM analysis is plotted with two-digit alleles to the left and four-digit alleles to the right defined by HLA imputation using SNP2HLA software with the Pan-Asian reference panel.

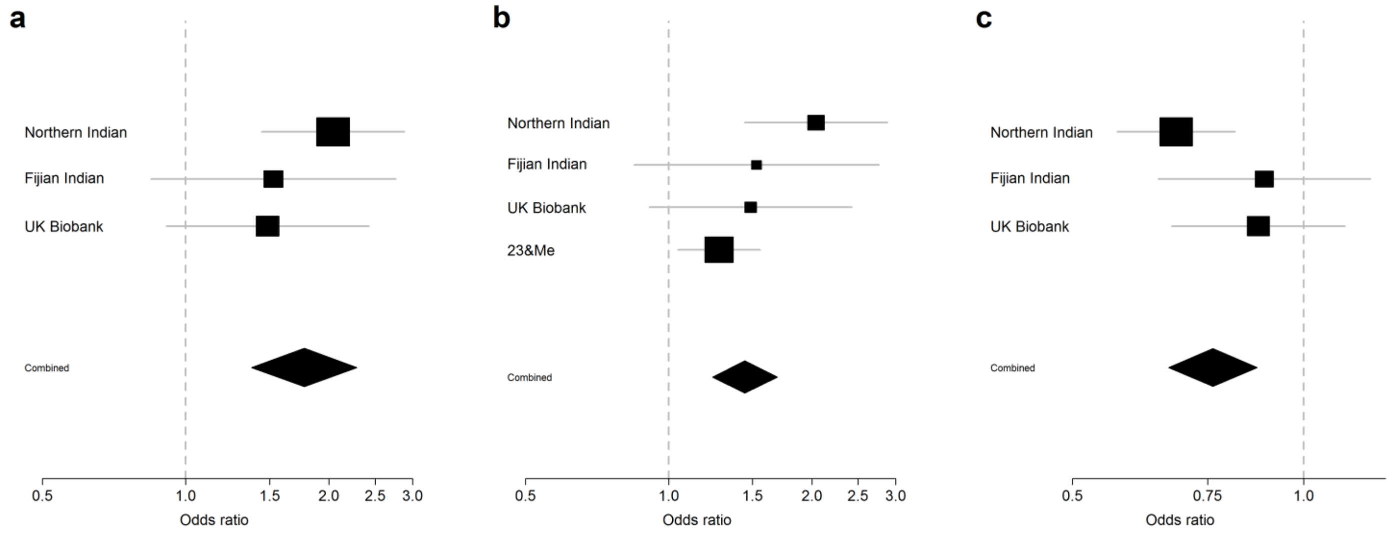

Supplementary Figure S6 | Forest plots of additional HLA alleles and amino acid variants following HLA imputation. (**a**) Presence of *HLA-DQB1**03:03. (**b**) Presence of *HLA-DQB1**03:03 in a combined analysis with the 23&Me data. (**c**) Presence of leucine at position -16 in *HLA-B*. For each analysis, the black squares centre on the odds ratio estimate from LMM on a logarithmic scale; the size of the square is proportional to the weight of the analysis. The horizontal line through each square corresponds to the confidence intervals. The black diamond centres on the combined effect estimate by fixed effects meta-analysis and stretches to the confidence intervals; the dashed line indicates no effect.

Supplementary Table S1 | Characteristics of participants in the UK Biobank replication case-control set.

	Mitral stenosis status	
	**Case**	**Control**
**Characteristic**	(N=150)	(N=1309)
Year of birth, mean (SD)	1945.3 (5.5)	1945.2 (5.3)
Age, years, mean (SD)	62.7 (5.5)	62.9 (5.3)
Age category, % (N)		
<60 years	21.3 (32)	22.1 (289)
60-64 years	29.3 (44)	31.2 (408)
65+ years	49.3 (74)	46.8 (612)
Sex, male, % (N)	27.3 (41)	28.3 (370)
Self-reported White British ethnicity, % (N)	100.0 (150)	100.0 (1309)
Deprivation, % (N)		
Least deprived third	24.7 (37)	30.0 (393)
Middle third	32.0 (48)	34.2 (448)
Most deprived third	43.3 (65)	35.8 (468)
Tertiary qualifications, % (N)	16.7 (25)	22.8 (298)
Body mass index, kg/m^2, mean (SD)	27.4 (5.1)	27.8 (5.1)
Height, cm, mean (SD)	165.3 (8.7)	165.3 (8.6)
Consumes alcohol >2 times/week, % (N)	32.0 (48)	40.0 (524)
Current smoker, % (N)	9.3 (14)	9.0 (118)
Vigorous physical activity <once/week, % (N)	56.0 (84)	38.0 (498)
Other self-reported conditions		
Rheumatic fever, % (N)	18.7 (28)	0.0 (0)
Aortic stenosis (rheumatic or non-rheumatic), % (N)	9.3 (14)	0.8 (10)
Source of case status		
Hospital admission, primary diagnosis, % (N)	42.7 (64)	
Hospital admission, secondary diagnosis only, % (N)	42.7 (64)	
Death record without prior hospital diagnosis, % (N)	2.0 (3)	
Self-report only, % (N)	12.7 (19)	

SD, standard deviation.

Supplementary Table S2 | Comparison of imputation R^2 across the two South Asian populations when performing HLA imputation using SNP2HLA with the T1DGC and Pan-Asian reference panels.

Variant	Position	Imputation R^2 (T1DGC)		Imputation R^2 (Pan-Asian)	
		Fijian Indian	Northern Indian	Fijian Indian	Northern Indian
HLA-DQB1 Thr185Ile	32737733	0.931	0.998	0.746	0.786
HLA-B Val-16Leu	31432889	0.963	0.969	0.938	0.970
*HLA-DQB1**03:03	32739039	0.976	0.999	0.778	0.803
*HLA-B**40:06	31431272	0.816	0.813	0.885	0.890
*HLA-A**02:11	30019970	0.880	0.951	0.974	0.956

Supplementary Table S3 | Association statistics from the South Asian and UK Biobank meta-analysis at HLA loci associated with susceptibility in Aboriginal Australians.

Variant	Position	BETA	SE	OR (95% CI)	*P* value
HLA-DQA1 Leu-16	32713236	-0.04	0.06	0.96 (0.84-1.08)	0.49
HLA-DQA1 Leu-16Met	32713236	0.05	0.06	1.05 (0.92-1.19)	0.48
HLA-DQA1 Ala69	32717257	-0.07	0.06	0.93 (0.82-1.06)	0.27
HLA-DQA1 Ala69Leu	32717257	0.05	0.06	1.05 (0.93-1.19)	0.45
HLA-DQB1 Asp38	32740723	-0.14	0.07	0.87 (0.76-0.99)	0.03
*HLA-DQA1**01:01	32716284	-0.02	0.08	0.98 (0.84-1.15)	0.81
*HLA-DQA1**01:03	32716284	-0.13	0.09	0.88 (0.74-1.04)	0.13
*HLA-DQA1**03:01	32716284	0.12	0.10	1.12 (0.92-1.37)	0.25
*HLA-DQB1**05:03	32739039	-0.04	0.11	0.97 (0.78-1.20)	0.75
*HLA-DQB1**06:01	32739039	-0.09	0.09	0.92 (0.76-1.10)	0.35

N.B. The previously reported *HLA-DQB1**04:02 and *HLA-DRB1**08:03 were not imputed in the present study.
SE, standard error; OR, odds ratio; CI, confidence intervals.
